# A biomimetic anthropomorphic leg phantom for wearable bioimpedance sensing in lymphedema monitoring

**DOI:** 10.2478/joeb-2026-0014

**Published:** 2026-09-11

**Authors:** Anna Bublex, Amalric Montalibet, Bertrand Massot, Claudine Gehin

**Affiliations:** INSA Lyon, Ecole Centrale de Lyon, CNRS, Universite Claude Bernard Lyon 1, CPE Lyon, INL, UMR5270, 69621 Villeurbanne,France

**Keywords:** PVA-C phantom, sensors validation, bioelectrical impedance spectroscopy, lymphedema, electrical conductivity, electrical permittivity, living tissue

## Abstract

This study aims to develop a reproducible, biomimetic, anthropomorphic leg phantom as a controlled platform for the development and evaluation of wearable sensors for low-frequency bioimpedance monitoring of lymphedema. As a proof of concept, a 20-cm multilayer model was developed to establish and characterise the materials and fabrication approach required for future full-scale sensor validation. Electrical and mechanical characterisation of polyvinyl alcohol cryogel (PVA-C) and PVA-based fat and muscle phantoms was performed. The influence of freeze-thaw cycle number and duration on electrical properties and Shore hardness was investigated to support large-scale multilayer fabrication. Muscle anisotropy was reproduced via directional structuring. Reproducibility of electrical properties and long-term stability of PVA-C hydrogels were evaluated under refrigerated, vacuum-sealed storage. A practical methodology for assembling multilayer anthropomorphic leg phantoms was implemented. Electrical properties remained stable across the investigated freeze/thaw conditions, whereas Shore hardness increased with the number of cycles. Two 20-cm-high, three-layer anthropomorphic leg phantoms, representing healthy and stage 2 pathological condition, were fabricated using 3D-printed moulds integrating bone, muscle, and fat. Interfacial bonding between the layers provided mechanical cohesion and electrical continuity. Bioimpedance measurements showed consistent differences between the healthy and lymphedema-mimicking configurations. The methodology enables fabrication and assembly of multilayer anthropomorphic leg phantoms with electrical stability and mechanical cohesion. This proof-of-concept provides a basis for the development of a full-scale anthropomorphic model and its subsequent integration with wearable bioimpedance sensing technologies for lymphedema monitoring.

## Introduction

Lymphedema, also known as lymphatic oedema, is a condition characterised by a decrease in lymphatic flow [1], leading to localised fluid retention and tissue swelling in one or more limbs [2]. It most commonly affects the limbs and can significantly alter limb volume and tissue composition. The disease affects about 250 million patients worldwide [3] and can be classified as either primary or secondary, depending on its underlying cause. Primary lymphedema is congenital, while secondary lymphedema is caused by a disruption or obstruction of the lymphatic system, which often occurs as a result of cancer treatment (removal of lymph nodes and radiotherapy), surgery or in connection with certain risk factors such as obesity [4]. The loss of mobility, pain and psychological distress associated with this condition have a devastating impact on patients’ daily lives [5].

Despite its significant impact on quality of life and long-term morbidity, lymphedema remains a pathology that is poorly addressed, insufficiently treated and inadequately monitored [2,4,5]. The clinical presentation of this condition encompasses swelling, tissue remodelling, and alterations in the composition of subcutaneous tissues, and is classified into four stages [1]:

- Stage 0: A latent or subclinical condition in which swelling is not yet evident despite impaired lymphatic transport. This stage may persist for months or years before overt oedema develops.
- Stage I: An early accumulation of protein-rich interstitial fluid that decreases with limb elevation. Pitting may occur.
- Stage II: A permanent accumulation of pathological solids, such as fat and proteins, with limb elevation alone rarely reducing tissue swelling. Pitting may be present; later in stage II, pitting may disappear as excess subcutaneous fat and fibrosis develop.
- Stage III: Lymphostatic elephantiasis, with pitting potentially absent and trophic skin changes such as acanthosis, fat deposition, and warty overgrowths.

Lymphatic dysfunction leads to the accumulation of interstitial fluid and an increase in tissue water content [6]. These features make it particularly well-suited to non-invasive biophysical monitoring techniques, such as bioimpedance spectroscopy (BIS). BIS is a technique for the assessment of the electrical properties of biological tissues, thus facilitating comprehension of their composition, structure, and functional state. Its extensive utilisation in the monitoring of both extracellular and intracellular fluid shifts renders it a particularly effective tool for the tracking of the progression and management of lymphedema [6]. Nevertheless, the validation of BIS in clinical and wearable contexts is hindered by the inherent variability of human physiology. Fluctuations in both inter- and intra-subject measurements over brief periods of time can have a substantial impact on the accuracy of measurements, thereby complicating the evaluation of sensor performance and the development of reliable monitoring systems.

In order to surmount these limitations, physical tissue models, more commonly referred to as phantoms, have been developed to simulate the electrical properties of biological tissues in a repeatable and controlled environment. These phantoms facilitate the standardisation of test conditions, ensure reproducibility of results, and eliminate ethical constraints associated with human or animal experimentation.

To date, lymphedema phantoms have mainly been developed to reproduce the anatomical, acoustic, or optical characteristics of lymphatic tissues for imaging applications [7,8,9]. However, to the best of our knowledge, no physical lymphedema phantom has yet been specifically developed to reproduce the electrical properties of the different tissues involved in lymphedema for the validation of bioimpedance-based sensors. This gap highlights the need for electrically characterised, multilayer tissue-mimicking models that can provide controlled and reproducible conditions for the development and validation of electrical sensing technologies for lymphedema monitoring. Such a multilayer approach is particularly relevant because the anatomical and structural changes associated with lymphedema are not uniformly distributed across tissue compartments. In this context, the present study investigates the development and electrical characterisation of tissue-mimicking materials and multilayer phantom models representative of lymphedematous legs at two different stages, with the aim of providing a reproducible platform for the development and evaluation of bioimpedance and related electrical sensors.

Traditionally, electrical phantoms have been fabricated using natural gelling agents or vegetable-based media filled with conductive particles [10,11,12]. While these materials are effective in mimicking electrical characteristics, they often lack long-term stability and exhibit poor mechanical robustness. In order to address these challenges, synthetic polymers such as silicones, elastomers, and filled gels have been proposed [13,14]. Among them, polyvinyl alcohol cryogel (PVA-C) has received particular attention due to its tissue-like mechanical properties, tunable stiffness, biocompatibility, and relatively low environmental impact [15]. PVA-C-based phantoms have been widely investigated for applications in which mechanical or morphological fidelity is required, including brain tissue phantoms [15] and ultrasound phantoms for anatomical modelling and training [16].

Although the use of PVA-C has primarily been motivated by its mechanical properties, its electrical properties can also be tailored through the incorporation of conductive additives. Previous studies have explored PVA-based multilayer phantoms for electrical applications, such as the two-layer phantom developed by Goyal et al., which included a NaCl-filled PVA-C layer [14]. However, the use of PVA-C as a common matrix for tissue-specific layers, together with the assembly of these layers through freeze/thaw processing, has received limited attention. In particular, freeze/thaw processing can promote interfacial bonding between adjacent PVA-C layers through the formation of hydrogen bonds between PVA chains, providing a means of assembling successive tissue-mimicking layers without introducing an additional adhesive interface.

In our previous work [17], different conductive additives were incorporated into PVA-C to investigate and tune its electrical properties. Building on this material-level characterisation, PVA-C-based formulations were then developed to mimic specific soft tissues, namely fat [18] and muscle [19]. While these studies demonstrated that appropriate tissue-mimicking materials could be developed individually, an important remaining challenge was their integration into a large, multilayered anthropomorphic structure while maintaining their electrical and mechanical properties.

The present study addresses this transition from material-level development to full-phantom development and characterisation. In this study, we present the design, fabrication, and characterisation of a 20-cm multilayer anthropomorphic leg phantom as a proof of concept for a future full-scale model. The phantom comprises one healthy leg and one lymphedema-mimicking configuration designed to reproduce selected morphological features associated with stage 2 lymphedema. The phantom was developed as a proof of concept to reproduce, as closely as possible, the electrical properties of human bone, muscle, and fat over the 1 kHz – 1 MHz frequency range, while providing controlled and reproducible mechanical characteristics. The pathological condition was simulated by increasing the thickness of the subcutaneous fat layer, thereby reproducing the anatomical changes associated with lymphedema. Each tissue layer was engineered using materials previously developed for tissue-mimicking applications: a PVA-C/beeswax emulsion loaded with activated carbon for fat [18], a PVA/agar hydrogel containing NaCl and graphite for muscle [19], and 3D-printed polycarbonate for bone. Custom 3D-printed moulds were used to ensure reproducible layer dimensions, anatomical proportions and interfacial alignment during fabrication.

To ensure its relevance for future integration with wearable monitoring systems, the phantom was designed according to a set of functional and structural specifications that should ultimately be achieved to meet the needs of lymphedema monitoring. The model is intended to reproduce realistic lower-limb anatomy and tissue proportions, allowing accurate electrode positioning and current distribution. It should be compatible with both dry and wet electrodes to accommodate various sensor technologies. Because the final monitoring device will operate under compressive conditions, the phantom should ideally withstand circumferential pressure without structural degradation. Long-term stability is another key requirement, ensuring consistent electrical and mechanical behaviour during storage and repeated measurements. The phantom is also intended to enable the representation of different lymphedema-related tissue configurations by adjusting tissue thicknesses, particularly that of the subcutaneous fat layer, while maintaining controlled and reproducible electrical and mechanical characteristics. Finally, reproducibility and environmental responsibility are fundamental objectives, encouraging the use of non-toxic, stable materials and low-impact fabrication processes. While not all of these constraints are fully achieved at this stage, they define the framework guiding the development and optimisation of the anthropomorphic phantom.

Beyond the development of the multilayer structure, particular attention was given to the influence of the freeze/thaw processing conditions on the properties of PVA-C-based materials. As PVA-C constitutes the hydrogel matrix of both the fat and muscle phantoms, its processing conditions may directly affect the properties and reproducibility of the final model. Therefore, the effects of the number of freeze/thaw cycles and freezing duration on the electrical properties and hardness of PVA-C, fat, and muscle phantoms were investigated. This assessment is particularly relevant for large-volume and multilayered phantoms, which may require repeated or prolonged freeze/thaw processing during fabrication. Furthermore, directional structuring was introduced during muscle gelation to investigate the reproduction of anisotropic electrical behaviour. Finally, the reproducibility of the electrical properties of the fat and muscle phantoms was evaluated, while the long-term stability of PVA-C hydrogels was assessed under refrigerated, vacuum-sealed storage conditions.

## Materials and methods

### Phantom fabrication

#### Materials

PVA was purchased from Sigma-Aldrich (fully hydrolysed, Molecular weight approximately 145,000 Da), Agar (Industrial agar, CONDALAB), NaCl (M=58.44 g/mol, APPLICHEM) from Dutscher, and graphite from NanoGrafi (micron powder, purity 99.9%, particle size 5–10 µm). Beeswax was sourced from Aroma-Zone (yellow natural beeswax) and activated carbon from SFB Laboratoires (Carbo 2000, powder). The polycarbonate filament used to print the bone was purchased from Ultimaker.

#### Fabrication process

Three main steps were considered in this work: (i) preparation and characterisation of the PVA-C samples to define the baseline properties of the material used for developing the muscle and fat phantom, (ii) development and characterisation of each anatomical layer of the phantom: bone, muscle and adipose tissue, (iii) fabrication of the complete anthropomorphic phantom.

**PVA-C:** The mixture was prepared by dissolving 15% w/w (mass percentage of the material relative to the mass of the solvent) PVA in deionised water at 90 °C under mechanical stirring for 30 minutes. Samples were then poured into a standard measuring cell and subjected to a freeze/thaw cycle (in this work, a cycle is defined as: 20 hours at −20 °C, followed by 8 hours at 23 °C).

**Preparation of fat layer:** The adipose tissue part of the physical model was developed using three materials: PVA as the mechanical base of the model, beeswax for its insulating properties and activated carbon as a dopant to bring the conductivity and permittivity of PVA-C as close as possible to the electrical properties of human adipose tissue. These materials were selected following the study presented in [18]. The final materials selected and their respective proportions in the mixture are shown in Table 1 below:

**Table 1:** Summarised composition of fat phantom.

Materials	Concentration (% w/w)
Demineralised water	Solvent
PVA	10
Beeswax	40
Activated carbon	1

**Figure 1:**
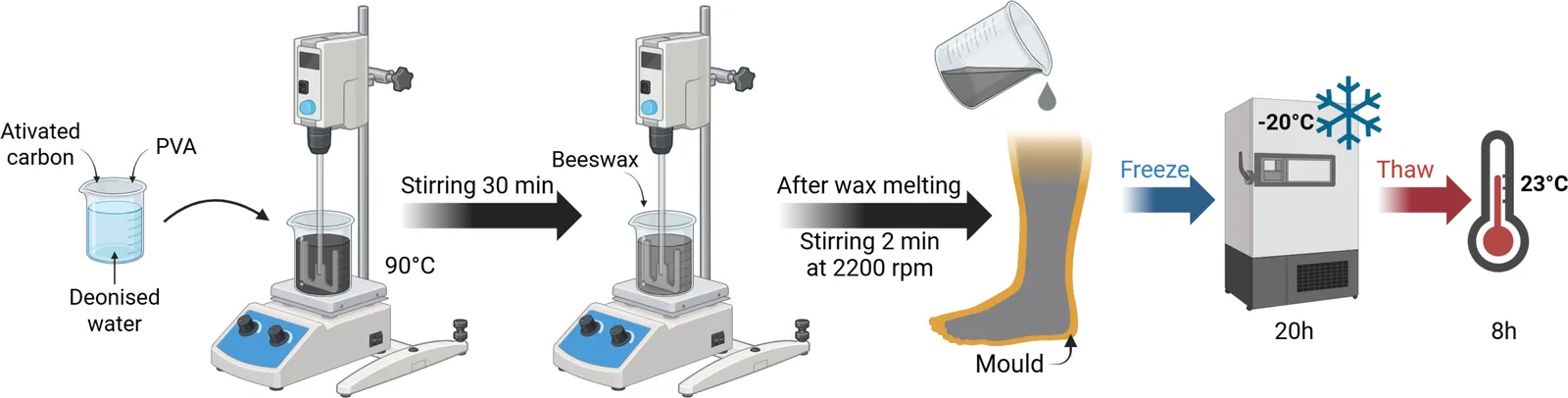
Fat phantom preparation steps. Created with Biorender.

The main steps in the fabrication of the adipose tissue phantom are illustrated in Figure 1. Initially, PVA and activated carbon were placed in a beaker containing demineralised water and mechanically stirred for 30 minutes at 90 °C. This step allowed the PVA to dissolve completely. Once the PVA had dissolved, beeswax was added to the mixture, while stirring and the temperature were maintained at 90 °C until the wax had completely melted. The mixture was then stirred at 2200 rpm for two minutes to form an emulsion. The resulting emulsion was transferred to a mould or a standard measuring cell before undergoing the freeze/thaw cycles.

**Preparation of muscle layer:** The muscle layer of the physical model was developed using four materials: PVA and agar as the basis for the mechanical strength of the model and graphite and NaCl to adjust the electrical properties. These materials were selected following the study presented in [19]. Their respective proportions in the mixture are shown in Table 2 below:

**Table 2:** Summarised composition of muscle phantom.

Materials	Concentration (% w/w)
Demineralised water	Solvent
PVA	15
Agar	1.5
Graphite	10
NaCl	0.3

**Figure 2:**
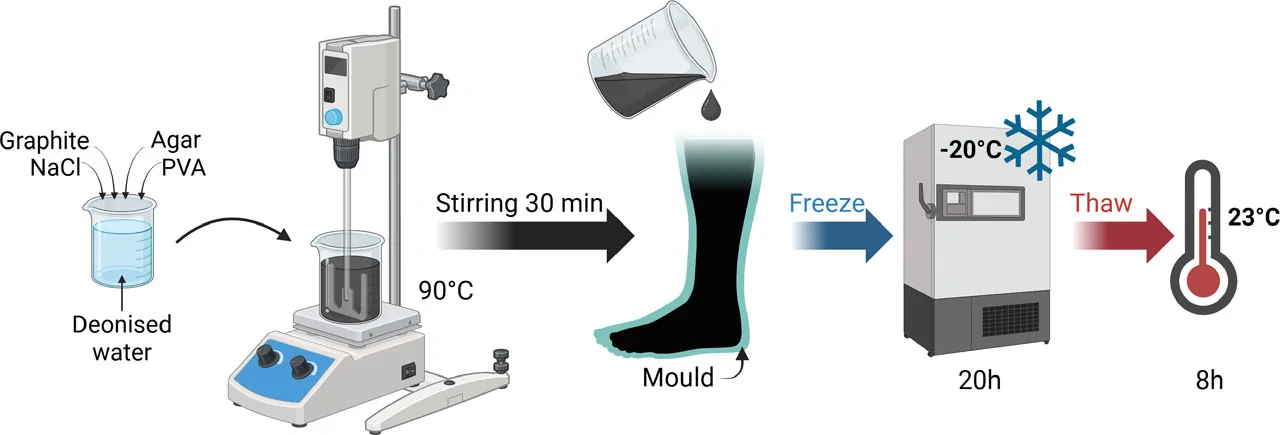
Muscle phantom preparation steps. Created with Biorender.

The main steps in the fabrication of the muscle phantom are illustrated in Figure 2. Initially, PVA, agar, graphite, and NaCl were placed in a beaker containing demineralised water and mechanically stirred at 90 °C for 30 minutes. This step allowed the PVA to dissolve completely. The resulting mixture was then transferred to a mould or a standard measuring cell before undergoing the freeze/thaw cycles.

**Preparation of bone:** The bones were fabricated using a 3D printing process with polycarbonate filament. To ensure anatomical accuracy, the leg model was created using Autodesk Fusion 360 based on an STL file derived from a CT scan (“Right Leg Bone Model 3D Printable STL File Converted from CT Scan – Extremity, Lower (Leg) – embodi3D.com”). The bones of the foot were removed and replaced with a straight rod to facilitate their positioning and fixation. A positioning notch was also added to the top of the moulds to ensure precise alignment during the different moulding stages. Given the central position of the bone within the phantom, its contribution to the overall electrical properties was considered negligible. In this model, the bone was therefore used primarily as a mechanical support.

**Fully assembled phantom:** To ensure anatomical consistency, the moulds of the leg models were designed from surface-based STL geometries provided by our project partner, the Textile Engineering and Materials research laboratory (GEMTEX). These geometries were developed as part of previous project work on the classification and modelling of lower-limb morphologies relevant to lymphedema [21]. They were used in the present study as simulated geometrical representations of the leg rather than as direct segmentations from clinical imaging.

Anthropomorphic moulds were designed from these geometries using Fusion 360 software (Autodesk). For the healthy and lymphedema-mimicking configurations, the bone and muscle geometries were kept identical. The muscle geometry was generated by geometrically contracting the healthy-leg surface geometry, and the same muscle geometry was subsequently used for both configurations. In order to mimic one of the morphological changes associated with stage 2 lymphedema, the thickness of the adipose layer was increased in the pathological configuration. This simplified approach focuses on changes in tissue thickness and does not reproduce the heterogeneous tissue remodelling and variations in fluid content that may occur in lymphedema. Thus, only the mould of the adipose layer differed between the two configurations.

**Figure 3:**
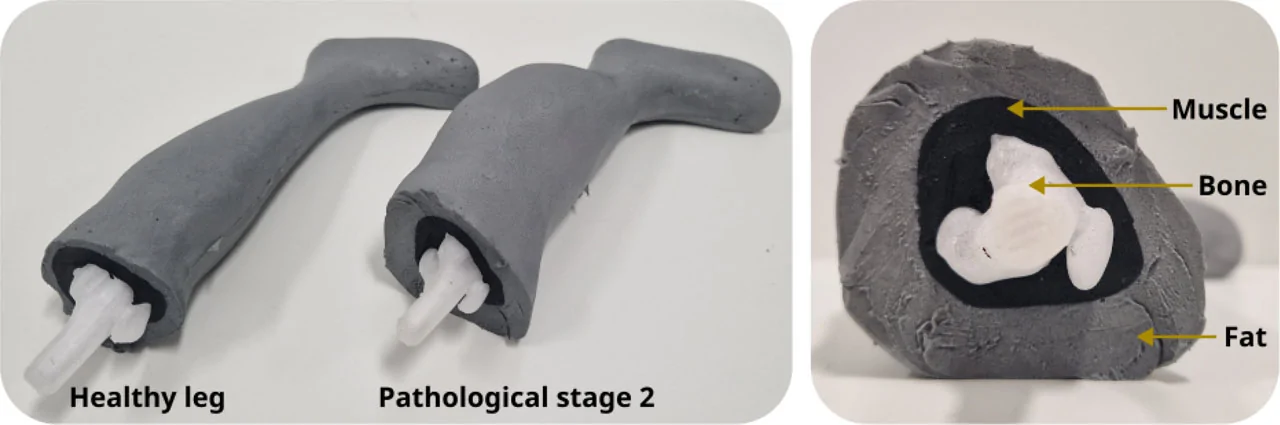
Healthy and pathological leg phantoms constituted by 3 layers: bone, muscle and fat.

Moulds for the development of two 20-centimetre leg configurations, one healthy and one lymphedema-mimicking, were selected for the proof of concept (see Figure 3).

**Figure 4:**
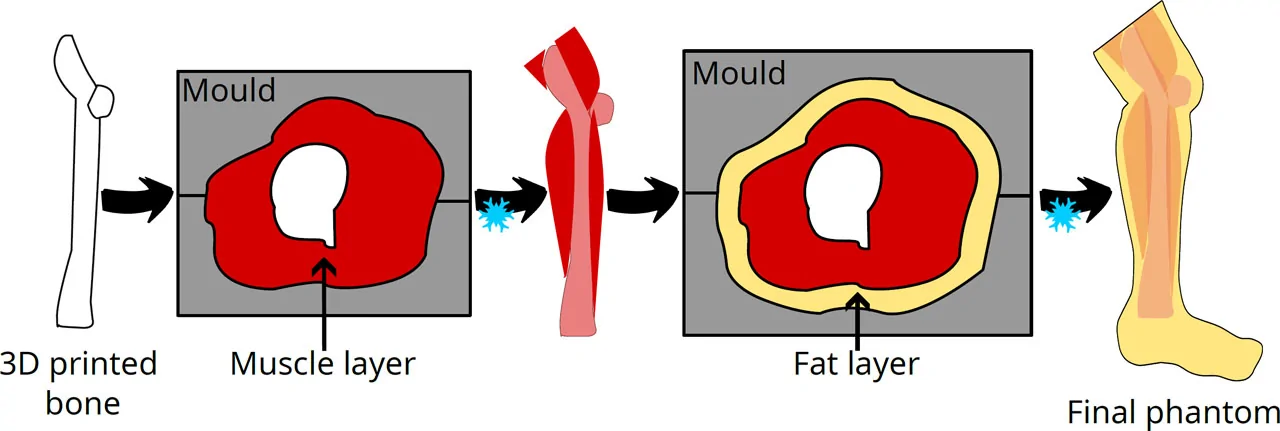
Fabrication steps of the anthropomorphic phantoms.

Figure 4 shows the steps involved in manufacturing an anthropomorphic physical leg model. The 3D-printed bone was positioned and fixed in the muscle mould before the muscle mixture was cast around it. The muscle mixture then underwent a freeze/thaw cycle before being removed from the mould and placed in the adipose tissue mould. The adipose tissue was then cast around the muscle phantom, followed by another freeze/thaw cycle. In total, the process involved two cycles for the muscle and one for the adipose tissue.

### Bioimpedance spectroscopy measurement

Bioelectrical impedance spectroscopy (BIS) is a noninvasive method used to determine the volumes of various body compartments, including total body water and extracellular water. BIS analyses fluid distribution and cellular membrane integrity by measuring impedance at various frequencies, providing insights into cellular health and hydration levels. Impedance is a generalisation of Ohm’s law for alternating current, expressed as a complex quantity as follows:

(1) Z=R+jX

where Z is impedance, R is resistance and X is reactance. The bioimpedance measurement instrument employed as a reference was the Multi-Frequency Impedance Analyzer (MFIA 5 MHz model), produced by Zürich Instruments. The device is capable of performing bioimpedance measurements across a frequency range of 1 mHz to 5 MHz, with a measurement range of 1 mΩ to 1 TΩ. In this study, measurements were conducted across a frequency range of 1 kHz to 1 MHz, with 100 data points acquired at logarithmic intervals.

#### Standard measuring cell – soft tissues characterisation

**Figure 5:**
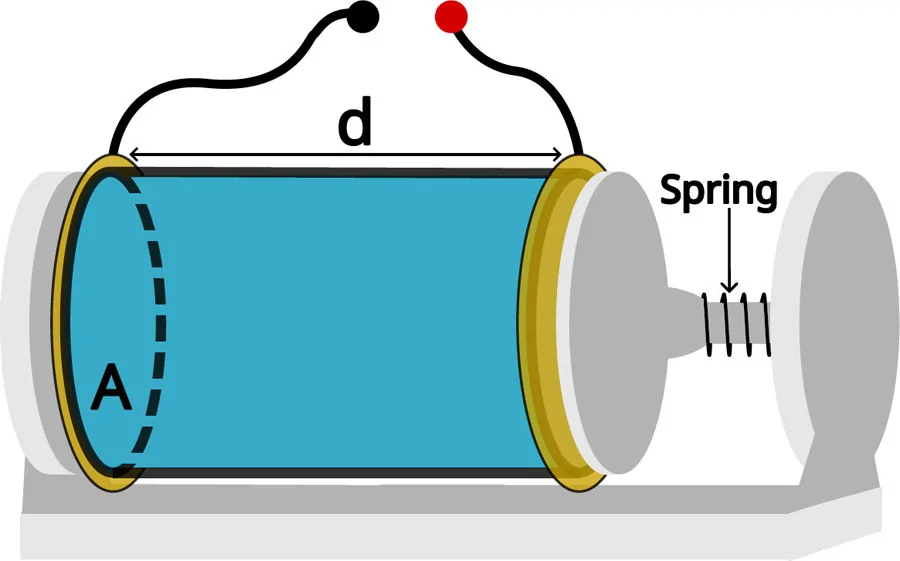
Schematic of a standard measuring cell. Length *d* = 50 mm, inner surface area *A* = 4.52 cm^2^.

The standard measuring cell was developed based on the model proposed in [22]. The cylinder employed for the containment of the samples was fabricated from transparent extruded polycarbonate, with a length of 50 mm and an inner diameter of 24 mm. The measurements were conducted using the standard measuring cell, as illustrated in Figure 5.

The electrodes under consideration were discs measuring 30 mm in diameter, with a gold-plated finish. The composition of these electrodes was as follows: a 35 µm copper base, a 5% nickel layer, and a 50 nm gold layer. The gold coating was of the ENIG 2U variety and was manufactured by JLCPCB. The support structure, which was designed in Autodesk Fusion 360 and 3D printed in PLA, incorporated a spring mechanism to ensure constant force application to the sample (see Figure 5).

The assessment of the conductivity and relative permittivity of a sample was conducted through the implementation of impedance measurements in a controlled geometry, utilising a two-electrode standard measuring cell. A comparison was then made between the obtained electrical properties and the literature values for living tissue [23]. To extract conductivity (*σ*) and relative permittivity (*ε*_r_) from bioimpedance measurements (Z), admittance (Y) was calculated following the methodology proposed by A. Ivorra [24]:

(2) $$Y\left(\omega \right)=\frac{1}{Z\left(\omega \right)}$$

With:

(3) $$Y\left(\omega \right)=G\left(\omega \right)+jB\left(\omega \right)$$

Where G is conductance and B is susceptance. Conductivity and relative permittivity were determined as follows:

(4) $$\sigma =\frac{G\left(\omega \right)}{K}$$

(5) $$\varepsilon_{r}=\frac{B\left(\omega \right)}{K\omega \varepsilon_{0}}$$

Where K is the cell coefficient, which in the case of an ideal electrode configuration (parallel and homogeneous geometry of the field lines) is expressed as *K = A/d*. As illustrated in Figure 5, *A* denotes the cross-sectional area, whereas *d* signifies the cylinder length. In this document, the terms ‘relative permittivity’ and ’permittivity’ are used interchangeably.

#### Four-electrode measurement on anthropomorphic phantom

**Figure 6:**
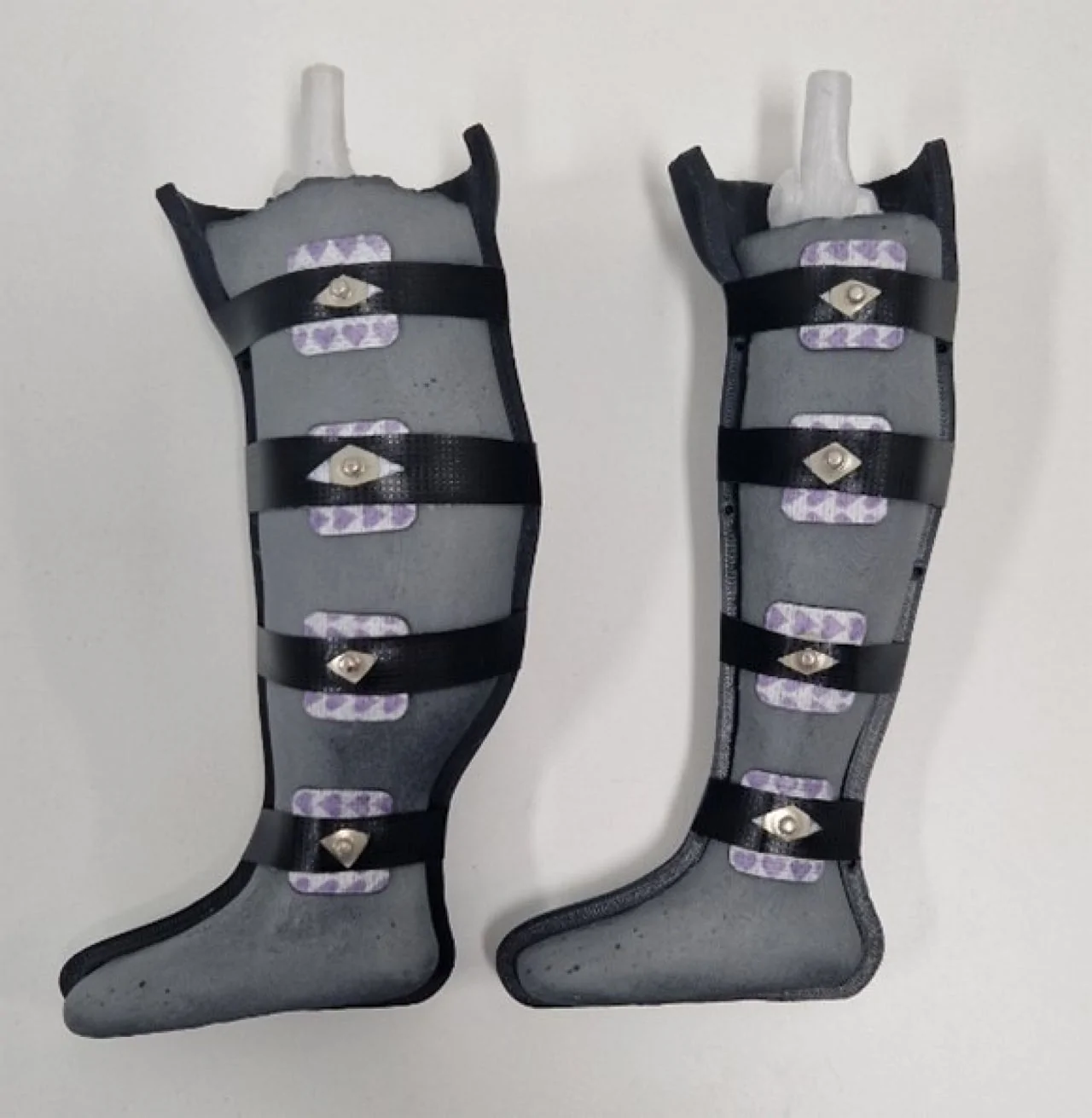
Anthropomorphic leg BIS measurement setup.

BIS measurements on the anthropomorphic leg were conducted using four pre-gelled electrodes (2.18 × 2.18 cm^2^, manufacturer: CONMED) arranged as shown in Figure 6. As the hydrogel electrodes did not adhere to the water-rich hydrogel, gaffer tape was used to secure them in place. The electrodes were spaced approximately 3 cm apart.

### Hardness measurement

The hardness measurements were conducted in accordance with the standards outlined in ASTM D2240, employing a Hildebrand Shore 00 hand-held durometer. For each measurement, the device was positioned vertically, and light manual pressure was applied to ensure uniform contact between the presser foot and the sample surface. In accordance with the recommendations outlined in the standard, values below 10 were excluded on the basis that they are considered unreliable when measured with a hand-held durometer. For each sample, ten measurements were taken at different locations and the arithmetic mean was calculated.

### Characterisation of soft tissue layers and PVA-C hydrogel

The stability, reproducibility and effects of freezing duration and the number of cycles were investigated using standard measuring cells.

#### Stability and reproducibility

To assess the temporal stability of the phantom and its usability over multiple days, impedance measurements were conducted on PVA-C hydrogel samples over consecutive days initially, and then once a week. The variability in the impedance modulus was assessed by calculating the relative deviation between the measurements taken on day X and the first day of measurement. This experiment was repeated over a period of 85 days. Between measurement sessions, the samples in standard measuring cell were stored in vacuum-sealed bags in a refrigerator at 4 °C. Each morning, the samples were removed from cold storage and measurements were performed once they had reached ambient temperature. The time required to conduct impedance measurements between 1 kHz and 1 MHz was approximately 2 minutes.

To evaluate the reproducibility of the fabricated phantom, six different batches of muscle phantom and three batches of fat phantom were prepared. Impedance measurements were taken over the frequency range from 1 kHz to 1 MHz.

#### Electrical properties and hardness of the soft tissue layer as a function of the number of cycles and of the freezing duration of the first cycle

This study aimed to deepen the understanding of PVA-C hydrogel properties, as well as those of muscle and adipose tissue-mimicking layers, with a view to their integration into multi-layered physical phantoms. Such models may involve specific fabrication constraints, including repeated freeze/thaw cycling for certain layers or the use of large-volume structures requiring extended freezing durations. The objective of this work was to characterise the electrical properties and Shore 00 hardness of PVA-C and soft tissue-mimicking hydrogels (muscle and fat) as a function of two key fabrication parameters: the freezing duration within a single cycle (ranging from 1 to 4 days) and the number of freeze/thaw cycles (from 1 to 4 cycles). Two sample types were investigated: (i) standard measuring cells for bioimpedance analysis, and (ii) 5 *×* 4 *×* 3.5 cm blocks for Shore 00 hardness testing.

**Figure 7:**
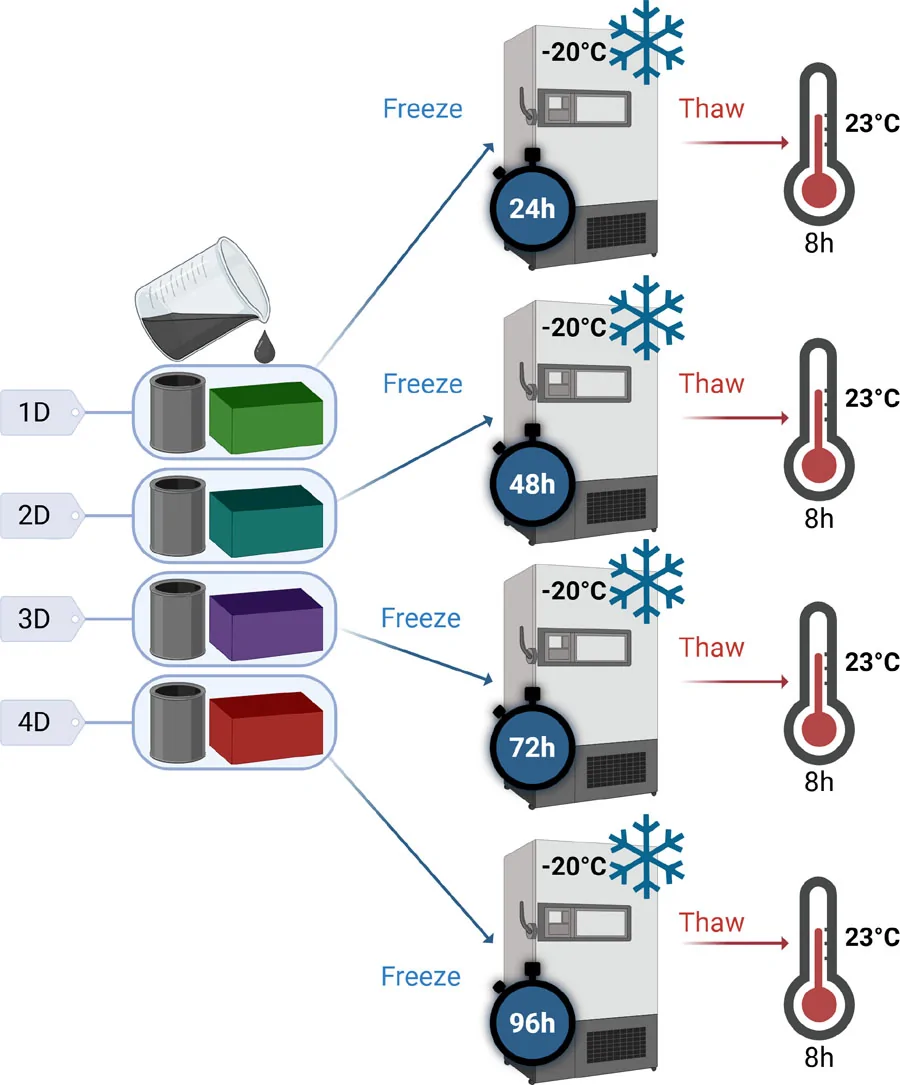
Preparation of samples for the study of the effect of cycle duration on the properties of PVA-C – ‘xD’ is used as a diminutive for ‘x’ days of freezing. Created with Biorender.

The influence of freezing duration was investigated with four groups of samples being prepared for the purpose of measuring electrical properties and Shore hardness over the course of four days (see Figure 7). The first group of samples was frozen for 24 hours, the second for 48 hours, the third for 72 hours, and the fourth for 96 hours. In each case, the samples were thawed for 8 hours to return to ambient temperature before the electrical properties and Shore hardness were measured.

**Figure 8:**
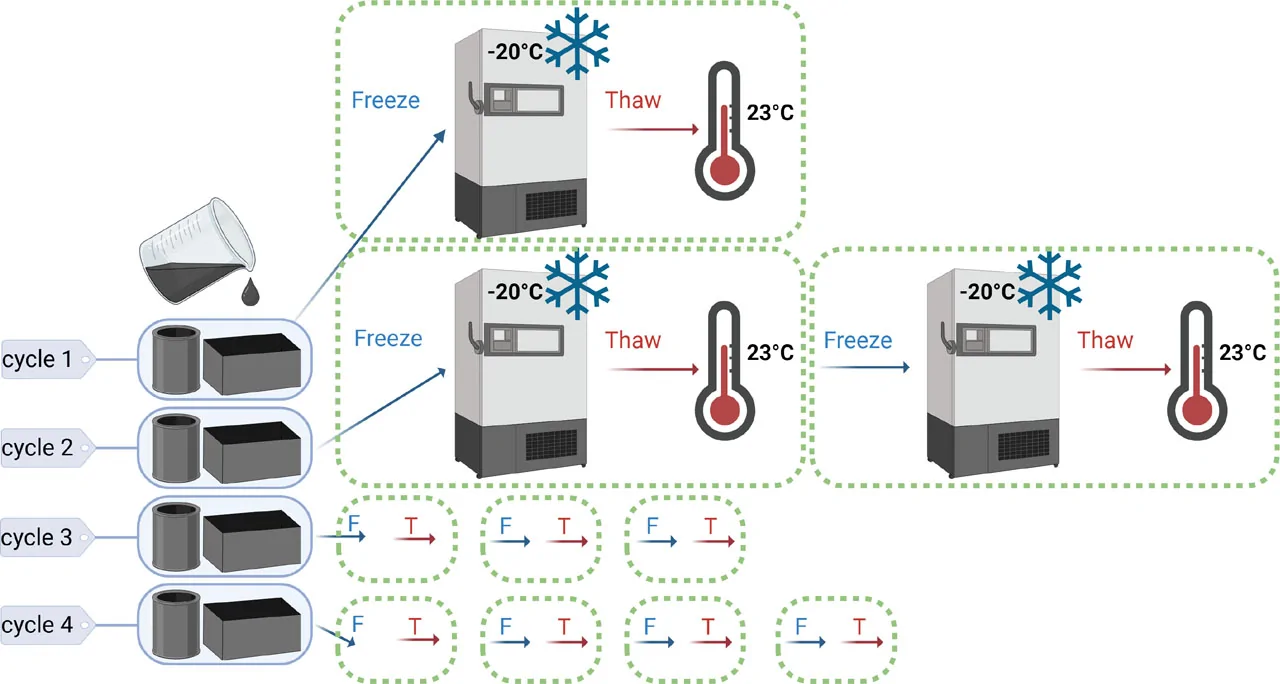
Preparation of samples for the study of the effect of the number of cycles on the properties of PVA-C. Created with Biorender.

For the study of the influence of the number of cycles, a single group of samples was prepared (see Figure 8). Each day, the samples were removed from the freezer and thawed at room temperature for 8 hours before electrical and Shore hardness measurements were performed. The samples were then returned to the freezer to undergo the next cycle. As the hydrogel progressively shrank during the successive cycles, a long tube with the same diameter as the measuring cell was used to allow samples of the appropriate dimensions to be cut at each measurement time point. The same sample was used for all Shore hardness measurements throughout the study.

#### Anisotropy of muscle

**Figure 9:**
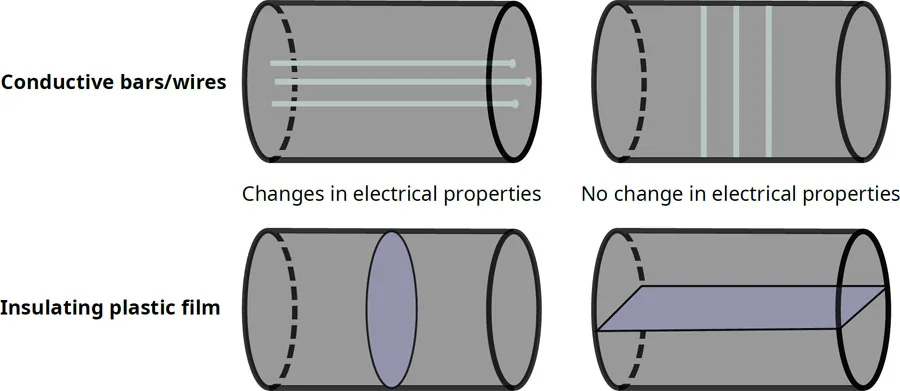
Methods for reproducing anisotropy in the muscle phantom.

In order to reproduce the variations in electrical properties related to muscle anisotropy observed *in vivo*, several experimental approaches were explored. As previously, all measurements were performed in standard measuring cells. Two main strategies were tested to induce anisotropy within the muscle model: the introduction of conductive elements and the addition of insulating materials. In the first approach, CONDUCTIB N 0.2 conductive wires (diameter: 0.33 mm, linear resistance: 0.2 Ω/m, TIBTECH), inserted using needles, and brass bars (diameter: 0.64 mm) were tested (see Figure 9 for the experimental configuration – 3 bars and 3 conductive wires were used for the measurements). Figure 9 illustrates the different methods used to induce anisotropy in muscle models.

These approaches were investigated as a material-level proof of concept to assess whether anisotropic electrical behaviour could be introduced into the PVA-C-based muscle material. Anisotropy was not incorporated into the final anthropomorphic phantom, but was investigated as a potential feature for future developments.

## Results and discussion

### PVA-C

PVA-C was first characterised independently to investigate the influence of freeze/thaw processing on its electrical properties and Shore hardness, as it constitutes the common hydrogel matrix of the fat and muscle layers.

#### Characterisation as a function of cycle parameters

**Figure 10:**
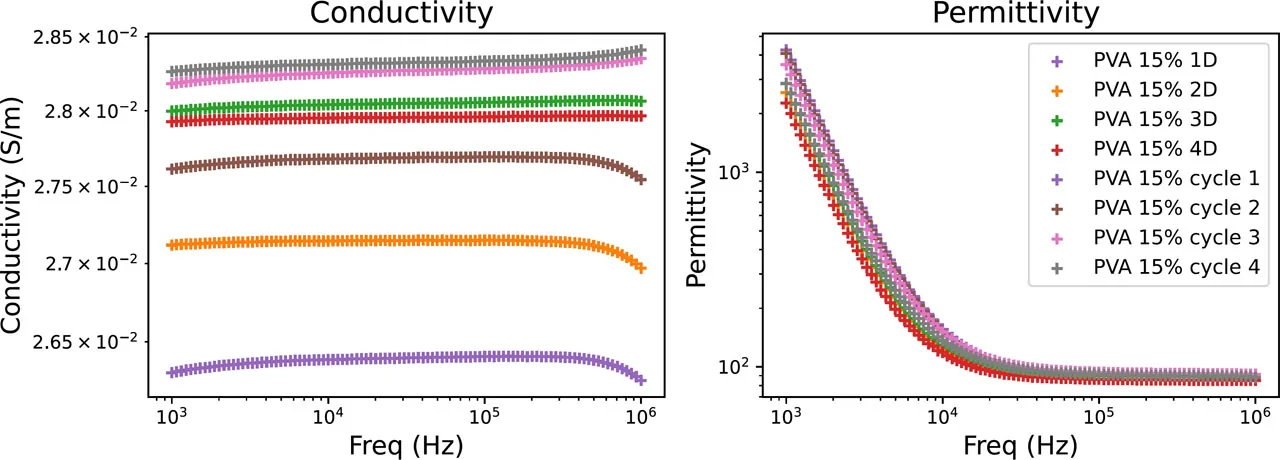
Electrical properties of PVA-C as a function of the duration of the freezing cycle and the number of cycles.

Figure 10 shows the evolution of the electrical properties of PVA-C hydrogel as a function of freezing time (from 1 to 4 days) and the number of freeze/thaw cycles (from 1 to 4 cycles), respectively. The results indicate overall stability in conductivity and permittivity, with no substantial variation observed as a function of these manufacturing parameters. The average coefficient of variation of the impedance modulus is 2.88% across the different freezing durations and 3.34% across the different numbers of cycles, indicating limited variability for this type of material and the intended applications. Furthermore, in all cases, the conductivity remains very low compared to that of human tissue, which limits the practical impact of these variations.

A plateau in permittivity is observed at high frequencies for the PVA-C hydrogel and fat phantom. This behaviour is associated with permittivity values approaching that of water (*ε*_r_ *≈* 80), consistent with the water-based composition of these materials. Similar behaviour has been reported for PVA-based hydrogels [17].

**Figure 11:**
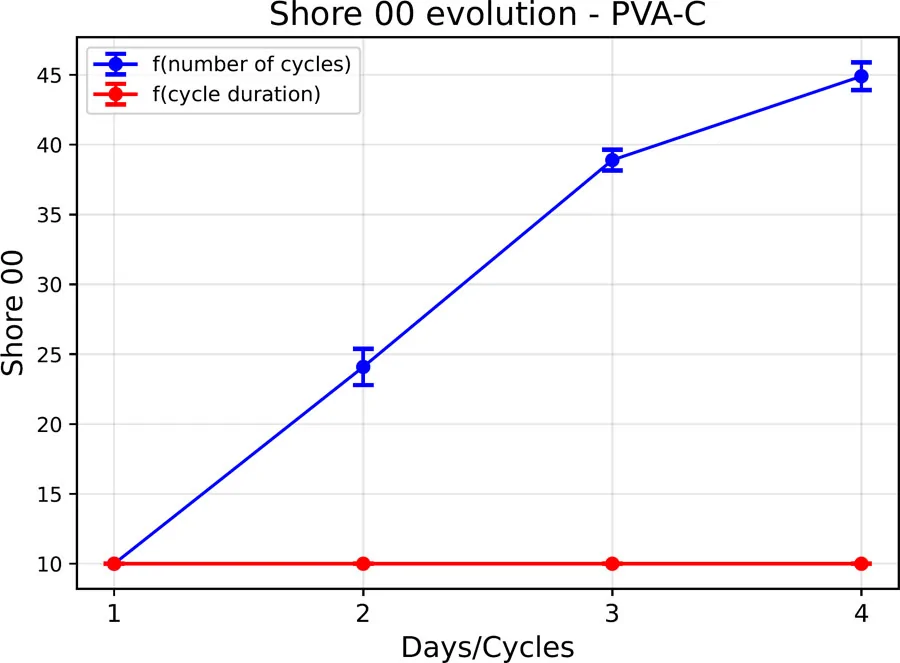
Shore 00 hardness evolution of a PVA-C sample.

The evolution of the Shore 00 hardness of the PVA-C hydrogel is shown in Figure 11. In accordance with ASTM D2240, values below 10 were not considered usable; they were therefore indicated by an arbitrary value of 10 on the graph in order to indicate their presence without interpreting them quantitatively. The error bars represent the standard deviation of the measurements. It appears that, whatever the duration of freezing (from 1 to 4 days), the hardness of the samples remains below this threshold. However, a gradual increase in hardness was observed with the number of freezing/thawing cycles. From the fourth cycle onwards, the hardness reaches around 45 Shore 00.

The increase in Shore hardness observed with increasing freeze/thaw cycle number can be related to the physical mechanisms underlying PVA-C formation. During freezing, ice formation induces local concentration of the PVA chains in the unfrozen polymer-rich phase, favouring intermolecular interactions and the formation of crystalline domains. These domains, together with hydrogen bonding between PVA chains, act as physical crosslinking points within the hydrogel network. Repeated freeze/thaw cycles can therefore progressively modify the degree of crystallisation and hydrogen bonding, resulting in changes in the final hydrogel properties, including increased hardness [25]. In contrast, the limited influence of freezing duration observed in the present study suggests that extending the freezing time does not substantially modify the resulting physical network under the investigated conditions.

This study contributes to a better understanding of the behaviour of PVA-C with a view to its integration into multilayer physical models, in which certain layers may be subjected to several freeze/thaw cycles, or in large-volume structures requiring prolonged freezing times. The results show that the electrical properties of the hydrogel remain stable overall, regardless of the duration of freezing or the number of cycles applied. On the other hand, the Shore 00 hardness is strongly influenced by the number of cycles, reflecting a gradual hardening of the material as the freezing/thawing cycles progress.

#### Evaluation of stability under refrigerated vacuum storage

**Figure 12:**
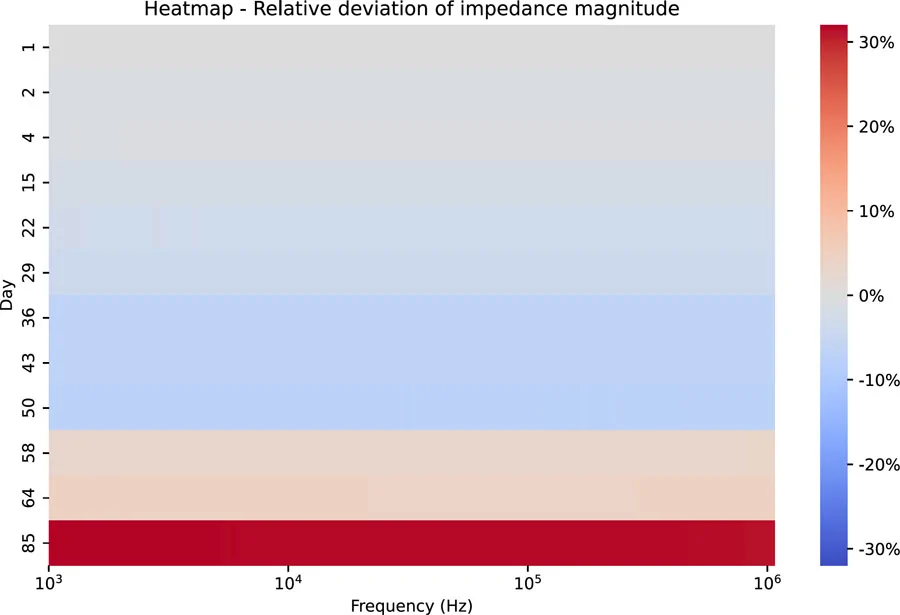
Heatmap – Relative deviation of the impedance modulus as a function of frequency – case of sample storage under vacuum in a refrigerator (4 °C).

**Figure 13:**
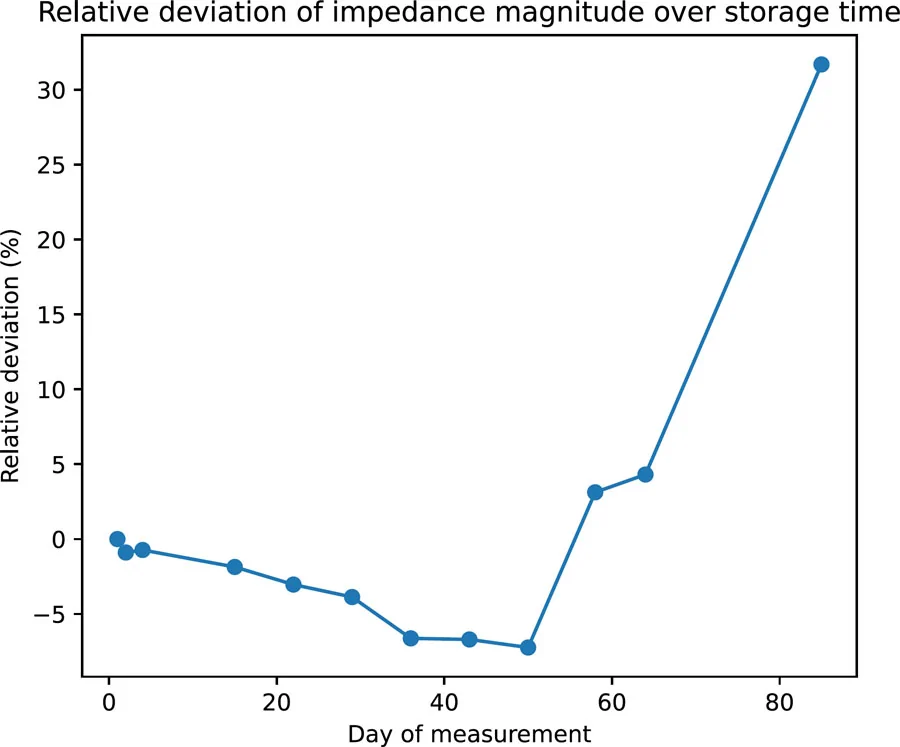
Average relative deviation of the impedance modulus over time – case of sample storage under vacuum in a refrigerator.

Figure 12 shows a heat map of the evolution of the relative deviation of the impedance module as a function of frequency and storage duration. Figure 13 shows the evolution of the average relative deviation (all frequencies combined) over time. The results indicate excellent stability during the first few weeks. Over the first four days, the relative deviation remains below 1%. On the 22^nd^ day, a deviation of 3% is observed, and stability is maintained below 10% until the 62^nd^ day. Beyond that, a more marked drift appears: the deviation reaches 30% on the 85^th^ day. The heat map reveals a generally homogeneous evolution across the entire frequency range and indicates that during the first 50 days, the impedance modulus tends to decrease, while after the 50^th^ day, the modulus tends to increase.

Several hypotheses can explain this late deterioration: sample shrinkage, loss of residual water, or accumulation of dust at the electrode-material interface. Nevertheless, these results show that the material remains electrically stable for more than two months, which is very satisfactory for laboratory use without the addition of preservatives.

This study has limitations, as the tests were carried out on a small sample under ideal experimental conditions: the measurements were brief and the sample was handled for a short period of time, then immediately returned to storage. These conditions do not necessarily reflect real-world use, where an anthropomorphic physical model could remain in the open air for several minutes or even hours while all the measurements are taken. Further evaluation under more representative conditions is still needed.

### Fat layer

**Figure 14:**
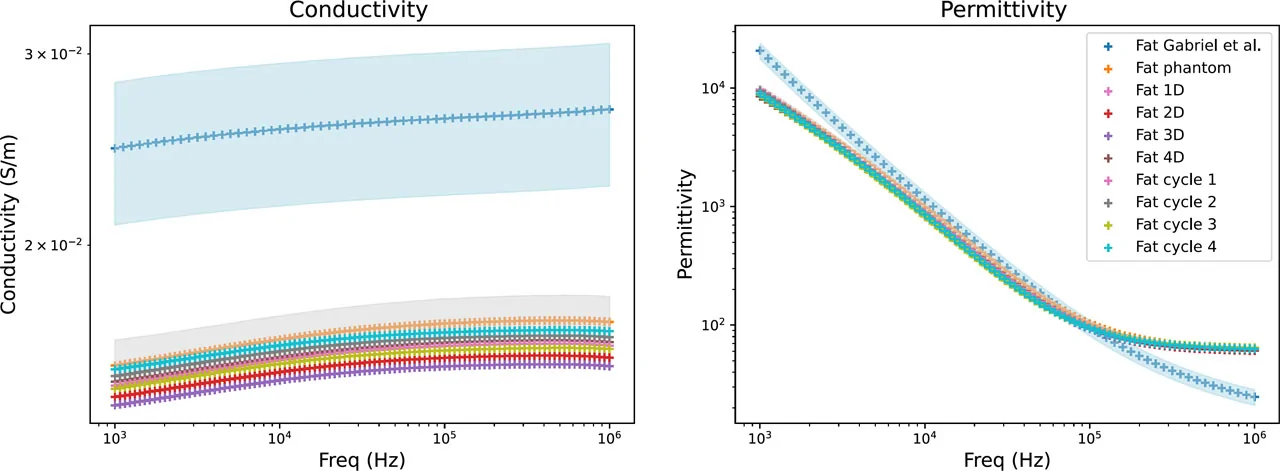
Electrical properties of fat phantom: literature data (blue), mean values of the developed fat phantom (orange), and their variability range (grey). The influence of freezing duration (Fat xD) and number of freeze/thaw cycles (Fat cycle x) is also shown.

Figure 14 shows a comparison of the electrical properties of the fat phantom (in orange) developed with data from the literature (in blue) by Gabriel *et al.* [23].

The permittivity of the developed adipose tissue phantom shows good agreement with the reference data between 10 kHz and 100 kHz, with a relative difference of −22.8% at 10 kHz and 0.4% at 100 kHz. Larger deviations are observed at 1 kHz and 1 MHz, with relative differences of −53.3% and 153.7%, respectively (see Table 3). In particular, the large deviation observed at 1 MHz is associated with the pronounced high-frequency permittivity plateau of the developed material, which is discussed in Section 3.1 for the PVA-C-based materials.

**Table 3:** Relative difference between the electrical properties of the developed fat phantom (Fat 1D data) and the reference values reported by Gabriel *et al.* [23].

Frequency	Conductivity	Permittivity
1 kHz	–39,53%	–53,30%
10 kHz	–38,72%	–22,77%
100 kHz	–38,28%	0,40%
1 MHz	–39,40%	153,73%

The conductivity of the developed phantom is systematically lower than the reference values, with relative differences of approximately −38 to −40% over the investigated frequency range. In developing this model, priority was given to adjusting the permittivity rather than the conductivity. Increasing the proportion of activated carbon could improve the agreement in conductivity, but would also modify the permittivity and potentially reduce its agreement with the reference values. The optimal proportion of activated carbon therefore depends on the specific requirements of the intended application.

#### Characterisation as a function of cycle parameters

Figure 14 shows the electrical properties of the adipose tissue model as a function of freezing duration and number of freeze/thaw cycles, respectively. The results indicate overall stability in conductivity and permittivity, with no substantial variation observed as a function of these manufacturing parameters. The average coefficient of variation of the impedance modulus is 2.29% as a function of freezing duration and 1.74% as a function of the number of cycles, which remains within an acceptable range of variability for this type of material and the intended application. Furthermore, the measured values show only limited deviations from the reference data (shown in blue in Figure 14), supporting the reproducibility of the manufacturing protocol.

**Figure 15:**
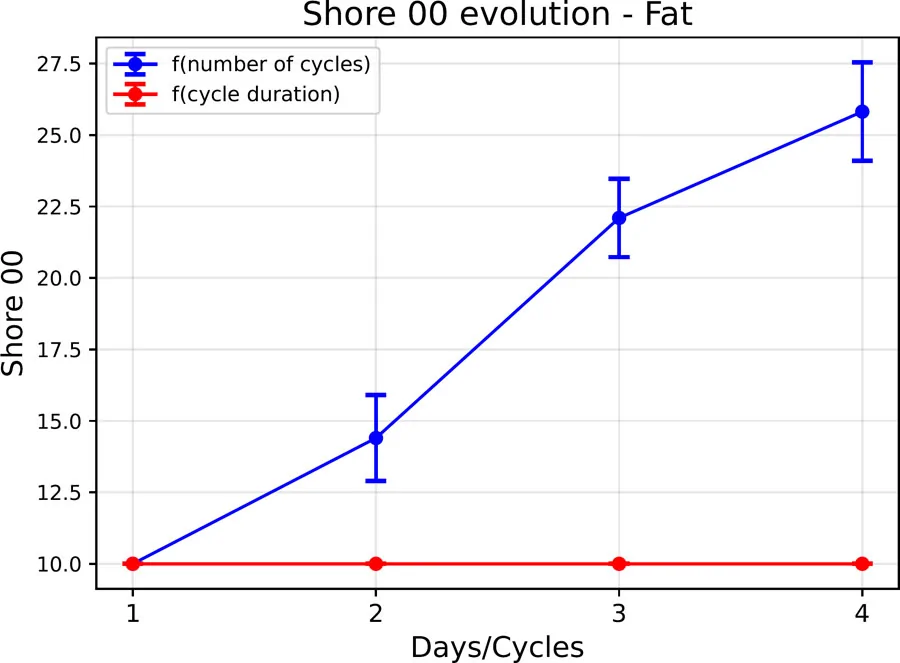
Evolution of the shore 00 hardness of the fat phantom as a function of the cycle parameters.

The evolution of Shore 00 hardness is presented in Figure 15. Measurements below 10 Shore 00 are considered unreliable and are therefore represented by a value of 10 in the figure for visualisation purposes only. Consequently, regardless of the freezing duration of the fat model samples, their hardness remains below 10 Shore 00. However, Shore 00 hardness increases with the number of freeze/thaw cycles, reaching 26 Shore 00 at cycle 4, a hardness lower than that of the PVA-C hydrogel studied previously.

These findings indicate that the electrical properties of the adipose tissue phantom remain largely unaffected by freezing duration or the number of freeze/thaw cycles. In contrast, Shore 00 hardness is primarily influenced by the number of cycles. Although this variation in hardness should be considered when defining the fabrication protocol, it does not affect the electrical properties relevant to the intended application. Therefore, these processing parameters are not expected to limit the development of the full-scale leg model.

#### Reproducibility

The reproducibility of the model was evaluated on a set of three samples prepared according to a strictly identical protocol, but using different preparation volumes. The average value (in orange in Figure 14) and the minimum and maximum values (in grey) are shown in Figure 14. The average coefficient of variation of the impedance module for these three samples is 4.89%. This level of variability demonstrates good reproducibility of the model, given the uncertainties associated with preparation (water evaporation), sample cutting and, above all, the emulsification of different volumes. In addition, the measured permittivity shows good agreement with the reference data between 10 kHz and 100 kHz, although the conductivity remains consistently lower than the reported values.

#### Limitation

The development of large fat phantoms can present certain challenges, primarily due to the difficulties encountered in maintaining emulsion stability during the processing of larger volumes. Tests carried out have produced homogeneous and stable emulsions up to a volume of 360 mL of aqueous solution (containing PVA, water and activated carbon) before the addition of melted wax. No attempts have been made beyond this volume, and the stability of the emulsion for larger preparations remains to be demonstrated. It is likely that the increase in volume complicates the rapid and effective homogenisation of the phases, making the process less reproducible. Specific optimisations, particularly in terms of mixing protocol or equipment, may be necessary to enable reliable scaling of the model.

### Muscle layer

**Figure 16:**
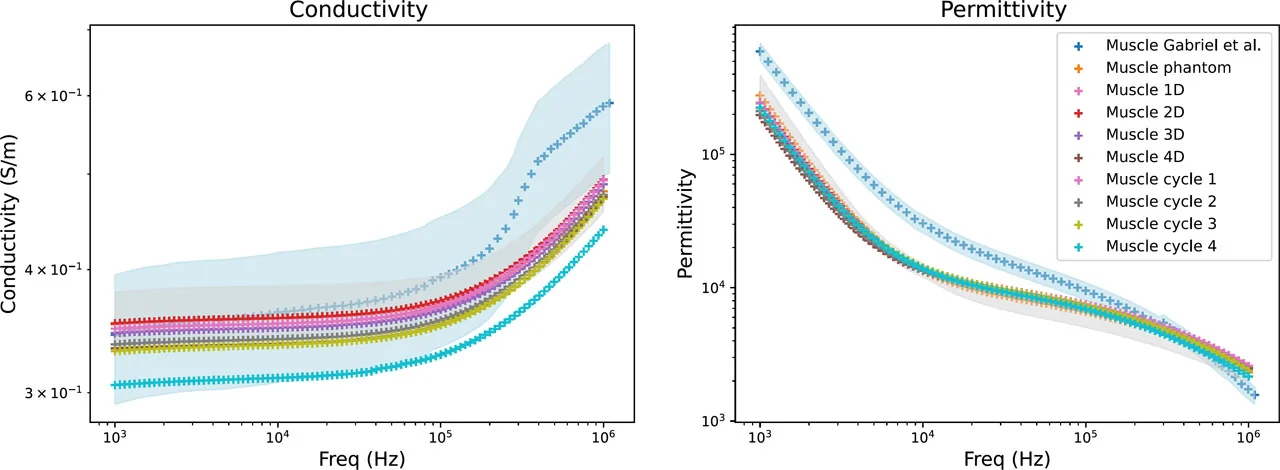
Electrical properties of muscle phantom: literature data (blue), mean values of the developed muscle phantom (orange), and their variability range (grey). The influence of freezing duration (Muscle xD) and number of freeze/thaw cycles (Muscle cycle x) is also shown.

Figure 16 presents a comparison of the electrical properties of the developed muscle phantom (in orange) with data from the literature (in blue) by Gabriel *et al.* [23].

The conductivity of the developed muscle phantom shows good agreement with the values reported by Gabriel *et al.* [23], particularly between 1 kHz and 300 kHz. Quantitative comparison (see Table 4) showed relative differences in conductivity ranging from 1.43% to −15.88% over the investigated frequency range. In contrast, larger deviations were observed for permittivity, with relative differences ranging from −59.32% at 1 kHz to 50.43% at 1 MHz. Nevertheless, the permittivity follows the same overall frequency-dependent trend as the reference data.

**Table 4:** Relative difference between the electrical properties of the developed muscle phantom (Muscle 1D data) and the reference values reported by Gabriel *et al.* [23].

Frequency	Conductivity	Permittivity
1 kHz	1,43%	−59,32%
10 kHz	−2,61%	−53,58%
100 kHz	−6,37%	−21,24%
1 MHz	−15,88%	50,43%

#### Characterisation as a function of cycle parameters

The electrical properties of the muscle model are presented as a function of freezing duration and number of freeze/thaw cycles in Figure 16. No substantial variation in the electrical properties was observed between the different freezing conditions. The average coefficient of variation of the impedance modulus across the different freezing durations was 2.39%, decreasing to 0.97% when considering freezing durations of less than three days. For the different numbers of freeze/thaw cycles, the average coefficient of variation was 5.15%, decreasing to 2.45% when considering three cycles or fewer. These results suggest that limiting the freezing duration to three days and the number of freeze/thaw cycles to three or fewer is preferable to preserve the electrical stability of the muscle model.

**Figure 17:**
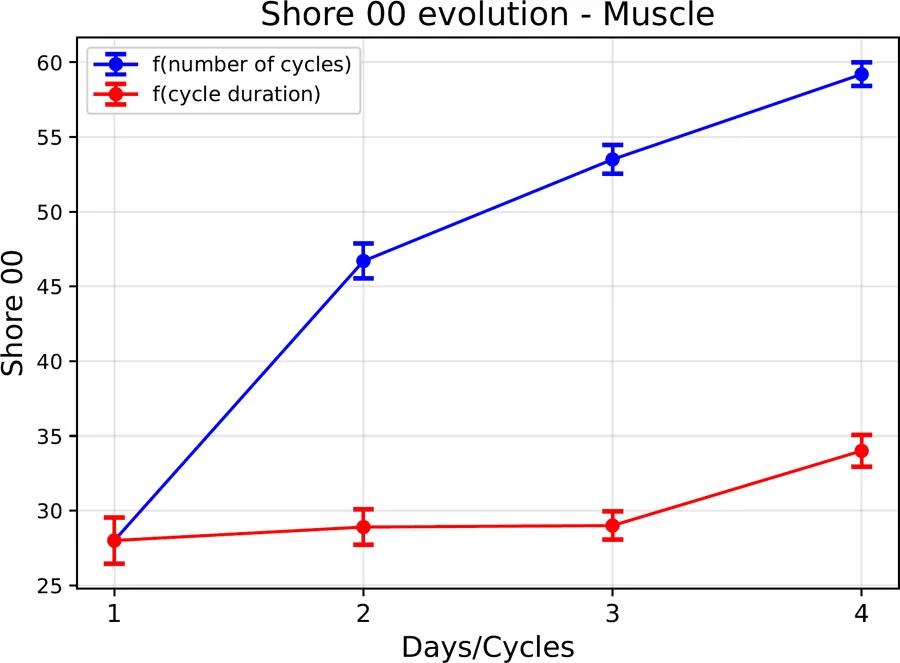
Evolution of the shore 00 hardness of the muscle phantom as a function of the cycle parameters.

The evolution of the Shore 00 hardness of the muscle model is shown in Figure 17. As was evidenced in preceding studies, an increase in the number of cycles results in an increase in the Shore 00 hardness, which in this instance reaches 60 Shore 00 at cycle 4, a value that exceeds that of the PVA-C hydrogel studied previously. A minor elevation in Shore 00 hardness was also detected in accordance with the duration of the freezing process. This trend was not previously identified in the studies conducted on the adipose tissue model or the PVA-C hydrogel. However, the hardness values for these two materials were below 10 Shore 00, which renders the measurements difficult to utilise. Consequently, the possibility of an increase in hardness, albeit not precisely measurable, cannot be discounted.

These findings suggest that the electrical properties remain largely unchanged for a freezing duration of three days or less, and for a limited number of freezing/thawing cycles of three or less. Conversely, the Shore hardness, while not constraining the development of this physical model, appear sensitive to the number of cycles undergone by the muscle phantom, and could also be influenced by the freezing duration. In order to limit the potential effects of these parameters on the Shore hardness and electrical properties, a maximum freezing duration of three days and a maximum of three cycles will be retained as thresholds for the manufacture of the muscle phantom.

#### Reproducibility

The reproducibility of the model was evaluated on a set of six samples prepared according to a strictly identical protocol, but using different preparation volumes. The average value (in orange in Figure 16) and the minimum and maximum values (in grey) are shown in Figure 16. The average coefficient of variation of the impedance modulus for these six samples is 5.29%. This level of variability demonstrates good reproducibility of the model, given the uncertainties associated with preparation (water evaporation) and sample cutting. Furthermore, the measured conductivity remains within the range reported by Gabriel *et al.* for muscle tissue between 1 kHz and 300 kHz, where the best agreement with the reference data was observed.

#### Anisotropy of muscle

**Figure 18:**
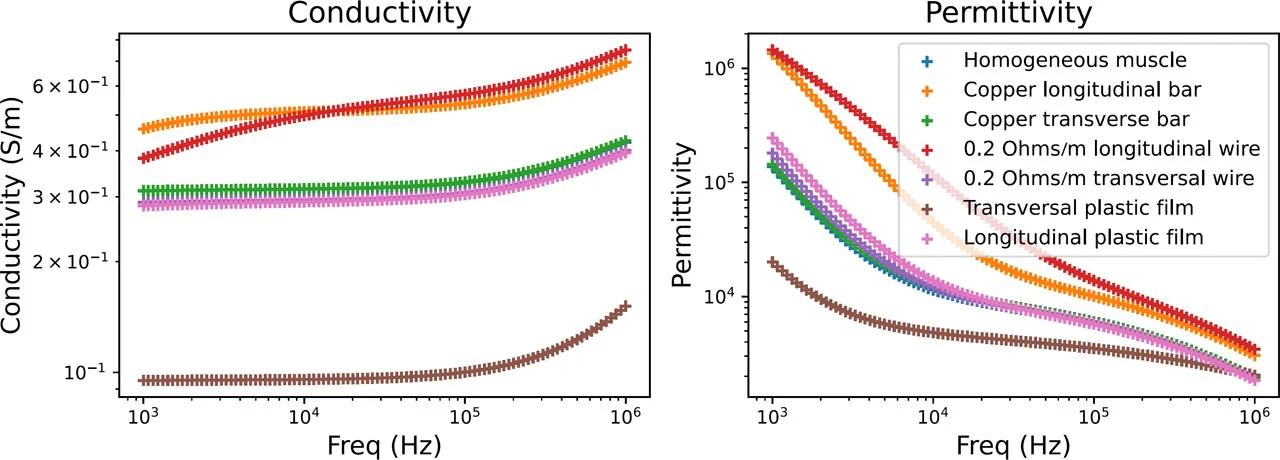
Comparison of electrical properties between the homogeneous muscle model and anisotropic variants.

The results indicate that when measurements are taken transversely to the conductive bars or longitudinally to the plastic film, the electrical properties are not significantly altered. However, the reverse causes notable variations, typical of anisotropic behaviour (see Figure 18).

**Figure 19:**
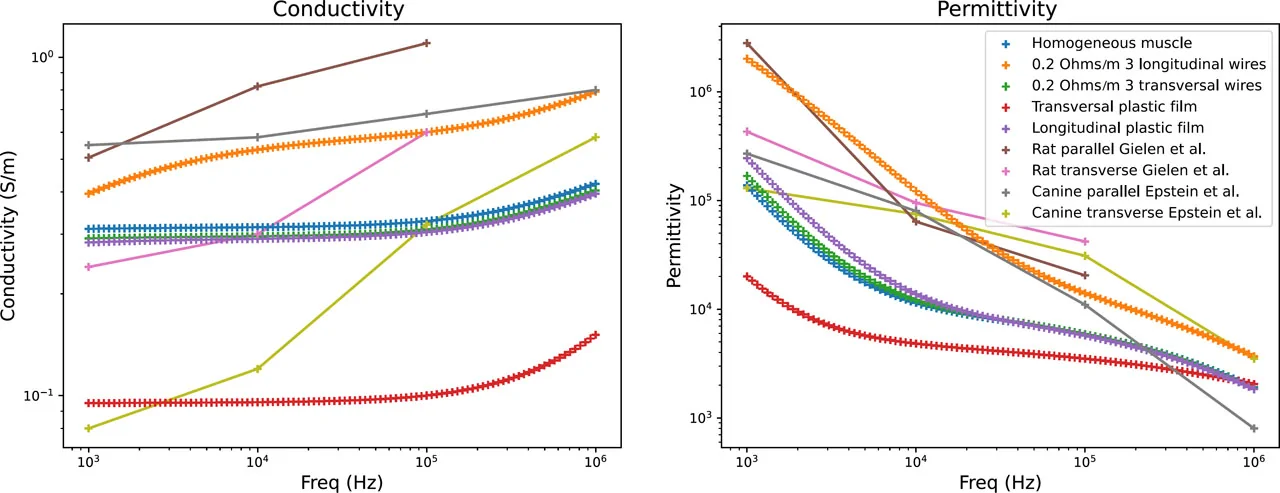
Comparison of the electrical properties of the homogeneous muscle model, anisotropic variants, and data from the literature.

These approaches could potentially be combined to tune the magnitude of the directional electrical behaviour according to the requirements of a future anisotropic muscle model. Figure 19 shows the results obtained with conductive wires and insulating films, compared with data from the literature. The observed frequency-dependent directional behaviour is consistent with the general anisotropic behaviour reported for muscle tissue.

According to Gabriel *et al*. [26], the anisotropy ratio between the transverse and longitudinal conductivity of muscle is around 10 or even higher. According to measurements by Kangasmaa *et al.* [27] anisotropy decreases with increasing frequency, with the ratio between lateral and longitudinal conductivity increasing from 37% to 64% in their study, conducted between 10 kHz and 1 MHz. In the anisotropy modelling proposed here, we obtain a minimum anisotropy ratio of 74% at 1 kHz and 50% at 1 MHz in the case of anisotropy created by conductive wires.

However, the conductive wires and insulating films used in this proof-of-concept do not reproduce the microstructural organisation of muscle fibres. In particular, the conductive wires introduce discrete conductive pathways that may produce artificial current distributions and therefore do not fully represent the anisotropic electrical structure of biological muscle. The measured anisotropy should consequently be interpreted as an effective anisotropy rather than a direct reproduction of muscle fibre organisation.

Anisotropy was not integrated into the anthropomorphic model developed in this paper. The anisotropy experiments were therefore performed as a separate proof of concept to assess whether directional electrical behaviour could be introduced into the PVA-C-based muscle material. Further work would be required to develop a more physiologically representative anisotropic structure and to integrate it into the multilayer anthropomorphic phantom.

### Anthropomorphic phantom

#### Adhesion between the layers of the physical model

Good adhesion between the different layers of the model is essential, not only to ensure its mechanical cohesion, but also to guarantee the continuity of its electrical properties. The presence of air or discontinuities at the interface can introduce artefacts during bioimpedance measurements. Furthermore, manufacturing defects, such as irregularities or discontinuities between layers, would reduce the repeatability of measurements.

The junction between the bone and the muscle is achieved by forming the muscle PVA-C directly around the bone during gelation. In practical terms, the 3D-printed bone is placed in the mould intended for the muscle, and then the mixture is poured around it. In its liquid state, the mixture closely follows the contours of the bone; the geometry, and in particular the space between the tibia and fibula, thus ensures mechanical support for the muscle around the bone once the gel has solidified. However, the hydrogel does not adhere directly to the bone material. The cohesion of the whole therefore relies mainly on the geometric interlocking and mechanical grip of the PVA-C around the bone, rather than on a true physico-chemical bonding between the hydrogel and the polymer constituting the bone.

**Figure 20:**
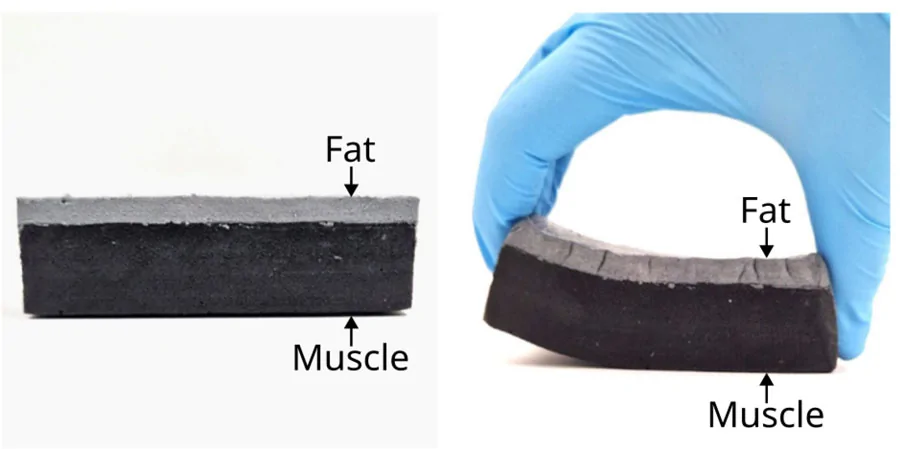
Connection between the muscle layer (black matter) and the layer of fat phantom (grey matter).

With regard to the juncture between the muscular and adipose layers: prior to the pouring of the fat layer, the muscle is subjected to a complete cross-linking process. A subsequent freeze/thaw cycle is then applied to the assembled layers. The two PVA-based materials form a stable interface, which may be facilitated by intermolecular interactions such as hydrogen bonding between PVA chains at the interface. As demonstrated in Figure 20, a pinch test reveals no visible separation between the two layers.

#### Bioimpedance measurements

**Figure 21:**
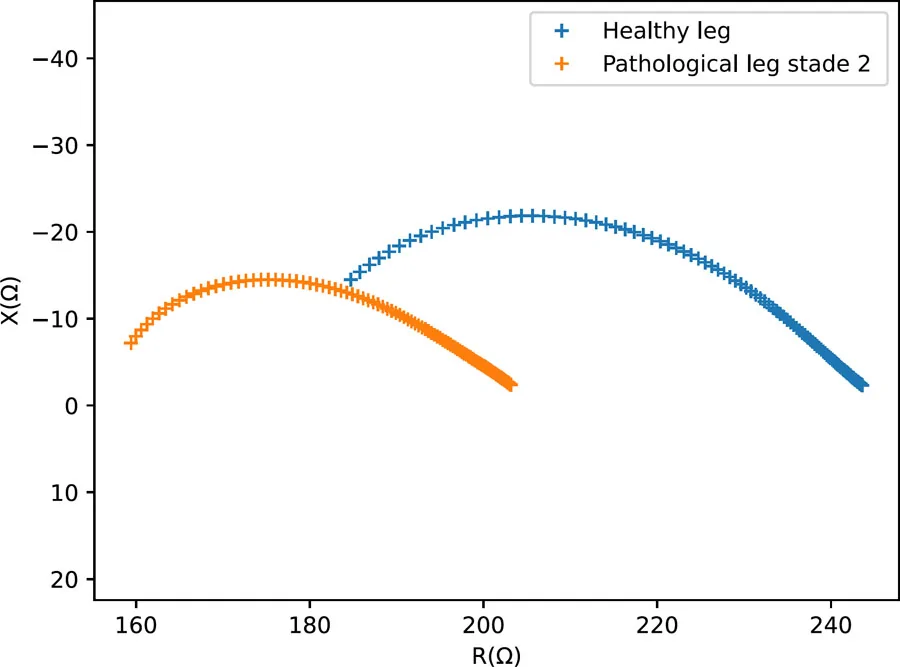
Nyquist diagram – Measurements on small-scale physical models representing a healthy and lymphedema-mimicking leg configurations.

Figure 21 shows the bioimpedance measurements performed using four electrodes on both the healthy and pathological leg. A shift in the arc of the circle is observed on the Nyquist diagram for the pathological model, with in particular a significant decrease in the real part of the impedance, indicating a lower equivalent series electrical resistance. These results are consistent with the expected behaviour. Indeed, a higher proportion of adipose tissue results in lower apparent resistance. However, these measurements should not be directly compared with in vivo measurements, given the small scale of the models (20 cm).

Although a skin layer has been initiated, this study focuses on the characterisation of the bone, muscle, and fat layers. The skin layer remains at the proof-of-concept stage and will require further development in future work. Nevertheless, the difference in electrical response between the healthy and lymphedema-mimicking configurations demonstrates the feasibility of detecting changes associated with the modified tissue geometry in the present proof-of-concept models.

## Conclusion

Physical models provide an ethical, stable, and reproducible alternative to in vivo experiments while enabling controlled modification of material properties. This study demonstrates the feasibility of manufacturing a multilayer anthropomorphic leg phantom with electrical properties representative of human tissues over the 1 kHz – 1 MHz frequency range, providing a platform for the future validation of electrical devices for lymphedema monitoring.

Each layer was independently characterised. Muscle was reproduced using 15% m/m PVA cryogel with 1% m/m agar, 10% m/m graphite, and 0.3% m/m sodium chloride, showing stable electrical properties and reproducible Shore hardness over up to three freeze/thaw cycles and freezing periods shorter than three days. Adipose tissue was developed from 10% m/m PVA, 40% m/m beeswax, and 1% m/m activated carbon, with stable electrical properties over four freeze/thaw cycles and four days of freezing. Bone was 3D-printed from PC filament, providing rigidity and thermal resistance.

The electrical characteristics of the soft-tissue phantoms were consistent with the targeted literature values. Shore hardness measurements provided a comparative assessment of the influence of freeze/thaw processing and demonstrated the reproducibility of the developed materials under the investigated conditions. PVA-C samples stored under vacuum in a refrigerator showed good stability over several months.

The multilayer assembly strategy, based on interlocking 3D-printed moulds and freeze/thaw processing, provided structural cohesion between the tissue-mimicking layers. The approach is adaptable to different anatomical regions, morphologies, and lymphedema-mimicking configurations using simple, non-toxic, and reproducible procedures. While soft tissue materials could still be optimised, this work represents a significant advance toward realistic, multilayer phantoms combining reliable mechanical performance with physiologically relevant electrical properties. Overall, this work establishes a reproducible proof-of-concept platform and provides a basis for the future development and validation of full-scale anthropomorphic phantoms for wearable electrical sensing technologies.

## Perspectives

Several avenues remain to further improve and validate the developed phantom. First, the mechanical characterisation of soft tissues should be extended. While Shore 00 hardness was investigated in the present study, Young’s modulus could not be reliably determined. Tensile tests and AFM measurements were limited by the softness of the hydrogels, sample handling difficulties, and water loss during testing. Elastography, which estimates mechanical properties from wave propagation, therefore represents a promising alternative for future characterisation. More generally, the present Shore hardness measurements provide a comparative indicator of the effect of freeze/thaw processing rather than a direct validation of mechanical equivalence with biological tissues.

Second, the temporal stability of the complete tissue-mimicking materials and assembled models warrants further investigation. In the present study, long-term stability was assessed only for PVA-C, while the stability of the doped fat and muscle formulations and of the assembled multilayer phantom was not systematically evaluated. The presence of agar, NaCl, graphite, and activated carbon may affect water retention, material degradation, and interactions between the different components. Dedicated ageing studies are therefore required to determine the storage lifetime and stability of the complete phantom. Mould growth was observed after two to three months under some storage conditions, highlighting the importance of appropriate handling and storage. Limiting prolonged exposure to air, using clean gloves and electrodes during measurements, and returning the phantom to vacuum-sealed refrigerated storage after short measurement sessions may help reduce contamination and water loss.

Further improvements should also aim to increase the physiological realism of the model. The pathological configuration developed in this study represents a simplified reproduction of selected anatomical features associated with stage 2 lymphedema. In particular, the model primarily reproduces the increase in subcutaneous tissue thickness, while variations in fluid content, fibrosis, and heterogeneous tissue remodelling are not explicitly reproduced. Future models could incorporate these additional features, as well as a skin layer with appropriate electrical and mechanical properties. Internal structures such as fasciae, vascular networks, and muscle anisotropy could also be introduced to better reproduce tissue heterogeneity and its influence on current distribution.

Finally, the approach should be transferred to a full-scale anthropomorphic model and evaluated under conditions representative of the intended application. The present 20-cm model was developed as a proof of concept to demonstrate the feasibility of the multilayer fabrication and electrical characterisation approach. Scaling up, particularly for the adipose layer, may require adapted emulsion protocols and equipment; the use of a lidded heating system during PVA-C preparation could help reduce evaporation and improve process consistency. The full-scale model will subsequently be integrated with the final wearable sensing device. This will enable further validation through electrode repositioning studies, inter-day repeatability measurements, measurements under circumferential compression, and comparison with *in vivo* BIS measurements.
